# Heart Fatty Acid Binding Protein and cardiac troponin: development of an optimal rule-out strategy for acute myocardial infarction

**DOI:** 10.1186/s12873-016-0089-y

**Published:** 2016-08-31

**Authors:** Joanna M. Young, John W. Pickering, Peter M. George, Sally J. Aldous, John Wallace, Chris M. Frampton, Richard W. Troughton, Mark A. Richards, Jaimi H. Greenslade, Louise Cullen, Martin P. Than

**Affiliations:** 1 University of Otago, Christchurch, New Zealand; 2 Lipid and Diabetes Research Group, Christchurch, New Zealand; 3 Emergency Department, Christchurch Hospital, Christchurch, New Zealand; 4 Canterbury Health Laboratories, Christchurch, New Zealand; 5 Cardiology Department, Christchurch Hospital, Christchurch, New Zealand; 6 Department of Emergency Medicine, Royal Brisbane and Women’s Hospital, Brisbane, Queensland Australia; 7 School of Medicine, The University of Queensland, Brisbane, Queensland Australia; 8 School of Public Health, Queensland University of Technology, Brisbane, Queensland Australia

**Keywords:** Heart Fatty Acid Binding Protein, Rule-out strategy, Acute myocardial infarction, High-sensitivity troponin, Accelerated diagnostic pathway, Multi-marker strategy

## Abstract

**Background:**

Improved ability to rapidly rule-out Acute Myocardial Infarction (AMI) in patients presenting with chest pain will promote decongestion of the Emergency Department (ED) and reduce unnecessary hospital admissions. We assessed a new commercial Heart Fatty Acid Binding Protein (H-FABP) assay for additional diagnostic value when combined with cardiac troponin (using a high sensitivity assay).

**Methods:**

H-FABP and high-sensitivity troponins I (hs-cTnI) and T (hs-cTnT) were measured in samples taken on-presentation from patients, attending the ED, with symptoms triggering investigation for possible acute coronary syndrome. The optimal combination of H-FABP with each hs-cTn was defined as that which maximized the proportion of patients with a negative test (low-risk) whilst maintaining at least 99 % sensitivity for AMI. A negative test comprised both H-FABP and hs-cTn below the chosen threshold in the absence of ischemic changes on the ECG.

**Results:**

One thousand seventy-nine patients were recruited including 248 with AMI. H-FABP < 4.3 ng/mL plus hs-cTnI < 10.0 ng/L together with a negative ECG maintained >99 % sensitivity for AMI whilst classifying 40.9 % of patients as low-risk. The combination of H-FABP < 3.9 ng/mL and hs-cTnT < 7.6 ng/L with a negative ECG maintained the same sensitivity whilst classifying 32.1 % of patients as low risk.

**Conclusions:**

In patients requiring rule-out of AMI, the addition of H-FABP to hs-cTn at presentation (in the absence of new ischaemic ECG findings) may accelerate clinical diagnostic decision making by identifying up to 40 % of such patients as low-risk for AMI on the basis of blood tests performed on presentation. If implemented this has the potential to significantly accelerate triaging of patients for early discharge from the ED.

## Background

Assessment of patients presenting to the Emergency Department (ED) with symptoms suggestive of acute coronary syndrome (ACS) represents a major clinical challenge [1], since the majority of patients do not have a final diagnosis of acute myocardial infarction (AMI) [2–4]. Several established accelerated diagnostic pathways (ADPs) utilizing risk scores together with sampling for cardiac troponin tests at presentation and at 2-or 3 h have been shown to accurately identify up to 40 % of patients as at low-risk of major adverse cardiac events (MACE) [5, 6]. However, earlier identification of low-risk patients at presentation alone could facilitate even earlier discharge, and thus promote further reductions in ED overcrowding and adverse patient outcomes [7–9].

A multi-marker strategy, combining hs-cTn with an early cardiac marker may improve sensitivity and increase the proportion of low-risk patients suitable for early discharge by allowing a higher troponin threshold to be utilized. Heart Fatty Acid Binding Protein (H-FABP), a highly myocardium-specific protein [10] and early rise marker of ACS [11], is a potential candidate. Rising H-FABP is detectable within at least 30 min following the onset of AMI, with concentrations that peak at approximately 6–8 h [12, 13] compared to 10–13 h for hs-cTnI and hs-cTnT [14]. In addition, the recent availability of a commercial immunoturbidimetric assay for H-FABP overcomes issues with previous point of care tests and ELISA-based assays, and means this biomarker can now be measured more reliably using the main laboratory platform [15, 16], concurrently with hs-cTn assays, and generate results within 20 min from blood sampling.

We assessed whether plasma H-FABP (by the new assay) at presentation, in patients attending the ED with chest pain, provides incremental value, in ruling out AMI, when used in combination with concurrently measured hs-cTnI (or hs-cTnT) and electrocardiography (ECG). In this feasibility study we aimed to identify the combination of H-FABP and hs-cTn that maximized the proportion of low risk patients correctly identified, whilst maintaining a high sensitivity for AMI. We have used the sensitivity threshold of >99 % to determine the biomarker thresholds, thereby ensuring a minimal false negative rate. Time frame as subgroups was also assessed as we hypothesized that H-FABP may add more diagnostic value in early presenters, given its early rise in AMI (compared to hs-cTn). It is recognised that <3 h following symptom onset that a low value of hs-cTn *alone* is insufficient to rule-out AMI (ESC 2015 guidelines).

## Methods

### Participants

We conducted a feasibility study on patients from the Christchurch hospital arm of the ADAPT study (ACTRN12611001069943) [3]. The protocol was approved by the Southern Health and Disability Ethics Committee, New Zealand (Ethics reference no: URA/07/06/048/ AM02), and all patients provided written informed consent. In brief, consecutive patients presenting acutely to the emergency department (ED) with symptoms suggestive of AMI, as per American Heart Association case definitions, were enrolled [17]. Exclusion criteria were any of the following: age <18 years; unable or unwilling to provide informed consent; a clear cause other than ACS for the symptoms; ST Segment Elevation Myocardial Infarction (STEMI); staff considered recruitment to be inappropriate (eg. receiving palliative treatment); transfer from another hospital; pregnancy; or inability to be contacted after discharge Patient assessment included blood sampling for contemporary TnI (c-TnI) at presentation and 6 to 12 h later in accordance with contemporaneous international guidelines [17, 18].

### Outcomes

The primary end point was AMI during initial hospital attendance. AMI diagnosis was based on evidence of myocardial necrosis, together with clinical features consistent with myocardial ischaemia (ischaemic symptoms, ECG changes or imaging evidence) [19]. Necrosis was diagnosed on the basis of a rising or falling pattern of the routine laboratory c-TnI (ARCHITECT c-TnI assay, Abbott), with at least one value above the 99th percentile (0.028 ng/mL) [20, 21]. Adjudication of the presence of AMI was performed independently by two cardiologists, and a third cardiologist made an independent final adjudication in cases of disagreement. Cardiologists were blinded to hs-cTnI, hs-cTnT and H-FABP results but had access to clinical records, ECGs, and serial non-high sensitivity c-TnI (ARCHITECT c-TnI assay, Abbott) results from routine care. Ischaemic ECG changes were defined using previous criteria [22–24].

### Assays

In addition to routine clinical tests, study samples were collected at presentation. EDTA and lithium heparin plasma were separated and frozen at −80 °C, within 2 h of sampling for later analysis using hs-cTnI and hs-cTnT, and updated H-FABP assays, respectively. Hs-cTnI concentrations were measured on the ARCHITECT STAT platform (Abbott Laboratories, Abbott Park, Illinois). The assay has a limit of detection (LoD) of 2 ng/L, 99th percentile among healthy subjects of 26 ng/L, and sex-specific 99th percentiles of 34 ng/L for men and 16 ng/L for women. Hs-cTnT concentrations were measured with the Roche Elecsys hs-cTnT assay (Roche Diagnostics Limited, Switzerland). The hs-cTnT assay has a LoD of 5 ng/L and a 99th percentile of the upper reference limit of 14 ng/L. H-FABP concentrations were measured with an immunoturbidimetric H-FABP assay (Randox Laboratories Limited, United Kingdom) on the ARCHITECT platform. The H-FABP assay can be performed concurrently with hs-cTnI and hs-cTnT assays on the same sample, and requires 20 min from the time of blood sampling to obtain a value. The 99th percentile of H-FABP assay in a healthy reference population is 3.6 ng/mL and LoD of 0.75 ng/mL [16]. No sex-specific differences in the 99th percentile concentrations of H-FABP have been shown [16].

### Optimum assay threshold determination

**Fig. 1:**
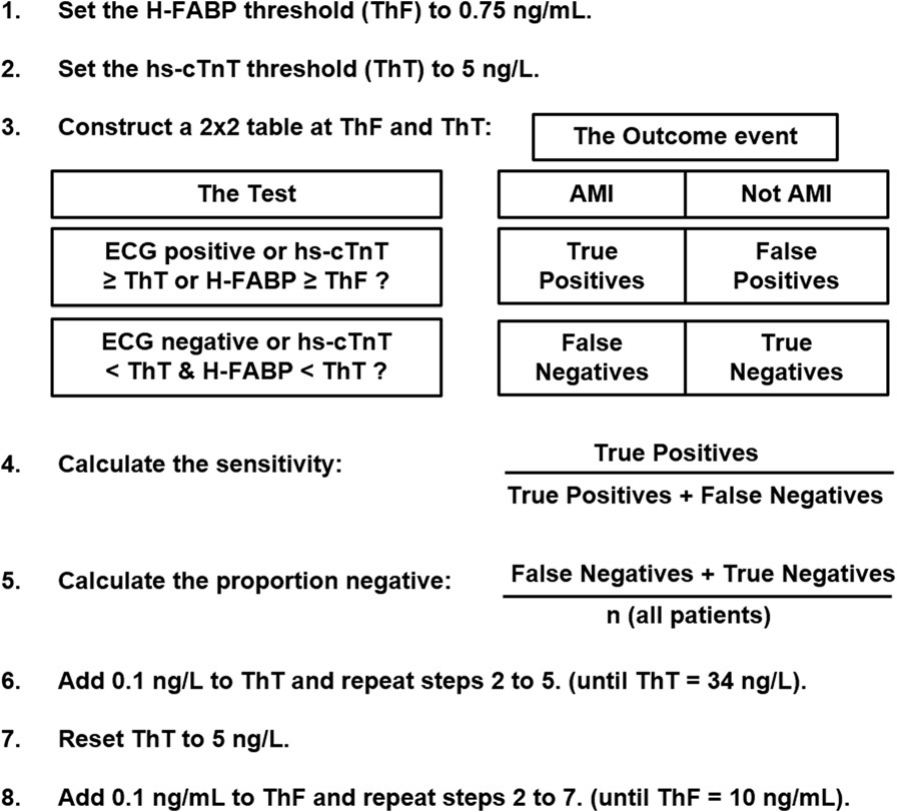
Methodology for calculating the each combination of H-FABP from LoD to 10 ng/mL (in steps of 0.1 ng/mL) and high-sensitivity troponin (hs-cTnI from the LoD to 34 ng/L in steps of 0.1 ng/L; hs-cTnT from the LoD to 14 ng/L in steps of 0.1 ng/L) the proportion low risk and sensitivity for AMI was determined in combination with the ECG

The primary goal was to determine the optimal combination of H-FABP with each hs-cTn assay which yielded the maximum proportion of low-risk patients whilst maintaining a minimum sensitivity for AMI of 99 %. We defined low-risk (of AMI), as those patients with a negative test, providing that the strategy has a sensitivity >99 %. The choice of 99 % sensitivity for AMI to determine the optimal marker thresholds was based on a survey suggesting this sensitivity, in ruling out major adverse cardiac events (MACE), is acceptable to most ED clinicians [25]. For each combination of H-FABP from LoD to 10 ng/mL (in steps of 0.1 ng/mL) and high-sensitivity troponin (hs-cTnI from the LoD to 34 ng/L in steps of 0.1 ng/L; hs-cTnT from the LoD to 14 ng/L in steps of 0.1 ng/L) the proportion low risk and sensitivity for AMI was determined in combination with the ECG (Fig. 1). This enabled a contour plot of sensitivities at sensitivities of 90 to 100 % in steps of 1 % to be drawn on the H-FABP - hs-cTn plane.

We also determined the proportion of patients with a negative test and sensitivity for AMI where a positive test was defined as ECG positive or (i) H-FABP ≥99th percentile, (ii) hs-cTn ≥ LoD, (iii) hs-cTn ≥99th percentile, (iv) hs-cTn or H-FABP ≥99th percentile, (v) hs-cTn ≥99th percentile or H-FABP ≥ the optimal Receiver Operator Characteristic curve (ROC) derived threshold (the threshold which maximized the combination of sensitivity and specificity in the group of patients in which the hs-cTn was <99th percentile), (vi) hs-cTnT ≥ the threshold which had >99 % sensitivity for AMI. Furthermore, ECG alone and together with H-FABP was also tested in combination with hs-cTnI only using sex-specific ≥99th percentile thresholds of 34 ng/L for men and 16 ng/L for women as a positive test. The potential of time from symptom onset to ED admission blood sampling to influence results was assessed by analysis of subgroups of >3 h, 3 to 6 h and >6 h from symptom onset to blood sampling. The estimated number of bed days saved per patient was calculated as a proportion of the total number of bed days for those patients with a negative test for the optimized thresholds for AMI-rule out, compared to the total number of bed days for all patients.

### Statistical analyses

Categorical data were expressed as numbers (percentages), and continuous data were expressed as mean (SD) or median (inter-quartile range (IQR)). The proportions of each of the AMI rule-out strategies-positive and –negative groups were reported along with the sensitivity and negative predictive values for AMI for each test. Confidence intervals are exact binominal 95 % confidence intervals. Subgroup group analysis was undertaken using the Chi-square test. Analyses were performed using SPSS version 22.0 (SPSS, Inc., Chicago, Illinois), or R [26] to determine the optimal thresholds.

## Results

**Fig. 2:**
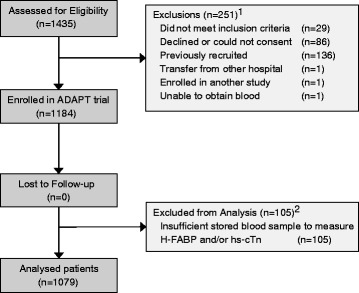
Consort diagram of study patients

Patients were enrolled consecutively between November 2007 and February 2011 at the Christchurch Hospital Emergency Department in Christchurch, New Zealand. 1184 patients were recruited. H-FABP, hs-cTnI and hsTnT results were available in 1079 patients (Fig. 2). Patients were predominantly male (59 %), aged 65 ± 13 years and presented a median (IQR) 6.2 (3.3–12.8) hours after symptom onset, and were a high cardiovascular risk population (Table 1). 1005 (93 %) patients were admitted to hospital for further investigation. An adjudicated diagnosis of AMI was made in 248 (23.0 %) patients.

**Table 1:** Characteristics of patient cohort at presentation and outcomes (*n* = 1079)

Age, yrs (mean / SD)	65	±13
Male, %	639	(59.2)
Systolic blood pressure, mmHg	145	±27
Diastolic blood pressure, mmHg	80	±14
Heart rate, bpm	74	±19
Weight, kg	82.0	±18.5
BMI, kg/m2	28.2	±5.6
Ethnicity		
Caucasian	993	(92.1)
NZ Maori	43	(4.0)
Pacific Island	10	(0.9)
Other	33	(3.1)
Risk factors at presentation		
Hypertension	662	(61.4)
Diabetes	172	(15.9)
Dyslipidemia	622	(57.6)
Current smoking	157	(14.6)
Family history of coronary artery disease	664	(61.5)
Prior medical history		
Angina	511	(47.4)
Acute myocardial infarction	324	(30.0)
Congestive heart failure	107	(9.9)
Cerebrovascular disease	135	(12.5)
Peripheral vascular disease	53	(4.9)
Coronary artery bypass graft	119	(11.0)
Percutaneous coronary intervention	265	(24.6)
Time of symptom onset to sample collection, h	6.2	(3.3–12.8)
Chest pain symptoms at presentation		
Pain at rest	957	(88.7)
Pain in past	535	(49.6)
Pleuritic pain	167	(15.5)
Radiation of pain	704	(65.2)
Diaphoresis	521	(48.3)
Outcomes		
ECG positive	181	(16.8)
STEMI	27	(2.5)
NSTEMI	221	(20.5)
Length of initial hospital attendance, h	50.1	(26.5–115.1)

Values are n (%) or mean ± sd, or median (lower quartile – upper quartile)

### Single biomarkers in combination with ECG

**Fig. 3:**
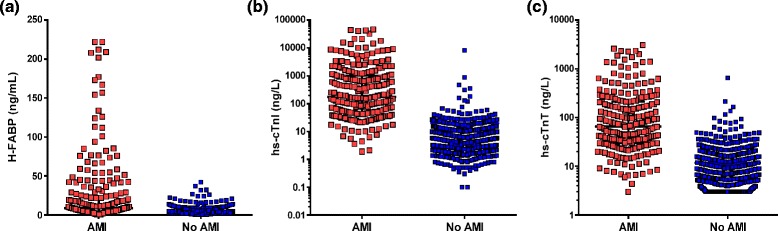
Scatter plots of **a** H-FABP, **b** hs-cTnI and **c** hs-cTnT concentrations at ED presentation in patients with and without an AMI diagnosis during index admission (*P* > 0.001 for the difference between groups for each biomarker)

H-FABP concentrations were greater in AMI than non-AMI patients, as were hs-cTnI and hs-cTnT levels (Fig. 3; *P* < 0.001). The median (IQR) H-FABP concentration in AMI patients was 8.7 ng/mL (4.5–24.5) and without AMI 3.4 ng/mL (2.2–5.3). H-FABP ≥99th percentile threshold (3.6 ng/mL) in combination with ECG had a sensitivity of only 90.3 % (95%CI 86.0 to 93.4) for AMI detection, with 24 false negatives. The median (IQR) level of hs-cTnI in AMI was 174 ng/L (126–222) and without AMI 4 ng/L (1–7), and for hs-cTnT in AMI was 66 ng/L (36–96) and without AMI 6 ng/L (3–9). Hs-cTnI performed similarly at ≥99th percentile threshold (26 ng/ml), Table 2. Hs-cTnT at ≥99th percentile threshold (14 ng/mL) performed slightly better, but still had a low sensitivity of 94.8 %), Table 4.

**Table 2:** Performance of H-FABP with hs-cTnI and ECG

Test		AMI (*n* = 255)	No AMI (*n* = 845)	Total (*n* = 1100)	Proportion Negative Test (%)	Sensitivity (%)	NPV (%)
Optimal combination^a^ : ECG positive or hs-cTnI ≥10 ng/L or H-FABP ≥4.3 ng/mL	Positive	246	392	638		99.2 (97.1 to 99.8)	
	Negative	2	439	441	40.9		99.5 (98.4 to 99.9)
Hs-cTnI 99th percentile threshold: ECG positive or hs-cTnI ≥26 ng/L	Positive	225	122	347		90.7 (86.5 to 93.7)	
	Negative	23	709	732	67.8		96.9 (95.3 to 97.9)
Hs-cTnI or H-FABP 99th percentile threshold: ECG positive or hs-cTnI ≥26 ng/L or H-FABP ≥ 3.6 ng/mL	Positive	244	442	686		98.4 (95.9 to 99.4)	
	Negative	4	389	393	36.4		99.0 (97.4 to 99.6)
Hs-cTnI 99th percentile with H-FABP ROC derived^b^: ECG positive or hs-cTnI ≥ 26 ng/L or H-FABP ≥ 3.1 ng/mL	Positive	245	511	756		98.8 (96.5 to 99.6)	
	Negative	3	320	323	29.9		99.1 (97.3 to 99.7)
Hs-cTnI threshold for >99.0 % sensitivity: ECG or hs-cTnI ≥ 3.9 ng/L	Positive	246	456	702		99.2 (97.1 to 99.8)	
	Negative	2	375	377	34.9		99.5 (98.1 to 99.9)

^a^Strategy that yielded the maximum proportion of low-risk patients whilst maintaining a minimum sensitivity for AMI of 99 %

^b^H-FABP ROC derived threshold which maximized the combination of sensitivity and specificity in patients negative for hs-cTnI and ECG

The optimal threshold for hs-cTnI in combination with ECG which had a sensitivity ≥99.0 % was 3.9 ng/L. 34.9 % of patients had a negative test. The optimal threshold for hs-cTnT in combination with ECG which had a sensitivity ≥99.0 % was 6.4 ng/L. 37.2 % of patients had a negative test.

The sensitivity of hs-cTnI ≥ LoD with ECG was 100 % (95 % CI 98.5 to 100.0), with 7.2 % of patients with a negative test. The sensitivity of hs-cTnT ≥ LoD with ECG was 99.6 % (95 % CI 97.8 to 99.9), with 30.0 % of patients with a negative test.

### H-FABP combined with hs-cTnI and ECG

**Fig. 4:**
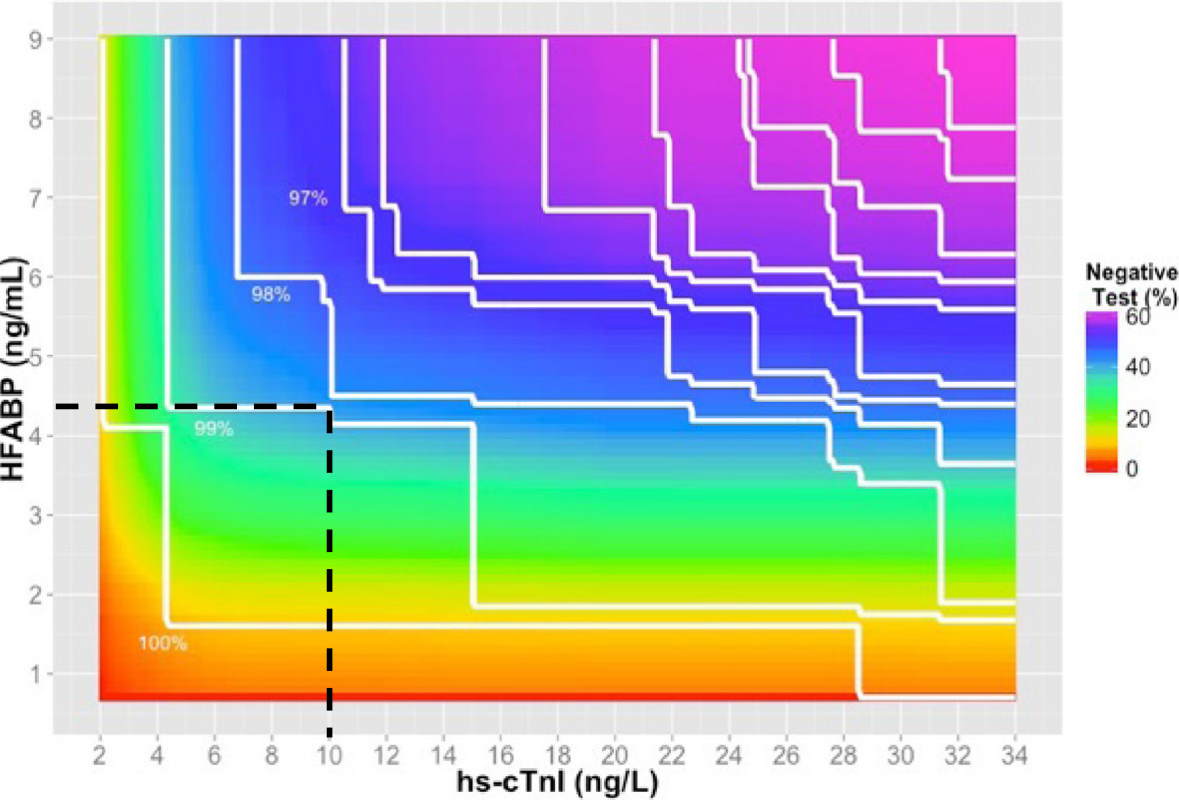
A. Contour plot of the sensitivities (*white lines*) and heat map of the percentage of negative test for AMI for any combination of H-FABP and hs-cTnI. Because multiple combinations of the two biomarkers can produce the same sensitivity we have plotted lines of constant sensitivity (eg 99 %). The colours represent the proportion of patients who have a negative test for each possible combination of biomarkers. *Red colours* represent few patients with negative tests, *purple colours* represent many patients with negative test. To focus on the high sensitivity areas we have limited the biomarker values displayed to ≤9 ng/mL for H-FABP and <34 ng/L for hs-cTnI. A negative test is defined when H-FABP and hs-cTnI concentrations are below the levels on the y and x axis respectively

The optimal combination that identified the maximum proportion of negative test patients (40.9 %) whilst maintaining >99 % sensitivity for AMI was H-FABP ≥4.3 ng/mL with hs-cTnI ≥10.0 ng/L (Fig. 4; Table 2). H-FABP and hs-cTnI at their respective 99th percentiles and ECG classified 36.4 % of patients as negative for AMI, with 4 false negatives (98.4 % sensitivity). The ROC derived optimal threshold for H-FABP when combined with hs-cTnI ≥99th percentile was 3.1 ng/mL. Applying this threshold reduced the number of false negatives by one (98.8 % sensitivity) and the overall proportion of negative test patients to 29.9 %. Sensitivity and Negative Predictive Value (NPV) were lower for the hs-cTnI sex-specific 99th percentile thresholds in combination with ECG when compared with the hs-cTnI 99th percentile threshold (26 ng/L) and ECG, but similar when combined with H-FABP (Table 3).

**Table 3:** Performance of H-FABP with hs-cTnI and ECG using sex-specific 99th percentile hs-cTnI thresholds

		AMI (*n* = 255)	No AMI (*n* = 845)	Total (*n* = 1100)	Proportion Negative Test	Sensitivity	NPV
Hs-cTnI 99th percentile threshold: ECG positive or hs-cTnI ≥16 ng/L (females) and ≥34 ng/L (males)	Positive	223	126	349		89.9 (85.5 to 93.1)	
	Negative	25	705	730	67.7		96.6 (95.0 to 97.7)
Hs-cTnI or H-FABP 99th percentile threshold: ECG positive or hs-cTnI ≥16 ng/L (females) or ≥34 ng/L (males) or H-FABP ≥ 3.6 ng/mL	Positive	243	442	685		98.0 (95.4 to 99.1)	
	Negative	5	389	394	36.5		98.7 (97.1 to 99.5)
Hs-cTnI 99th percentile with H-FABP ROC derived^a^: ECG positive or hs-cTnI ≥16 ng/L (females) or ≥34 ng/L (males) or H-FABP ≥ 3.1 ng/mL	Positive	245	511	756		98.8 (96.5 to 99.6)	
	Negative	3	320	323	29.9		99.1 (97.3 to 99.7)

^a^H-FABP ROC derived threshold which maximized the combination of sensitivity and specificity in patients negative for hs-cTnI and ECG

### H-FABP combined with hs-cTnT and ECG

**Fig. 5:**
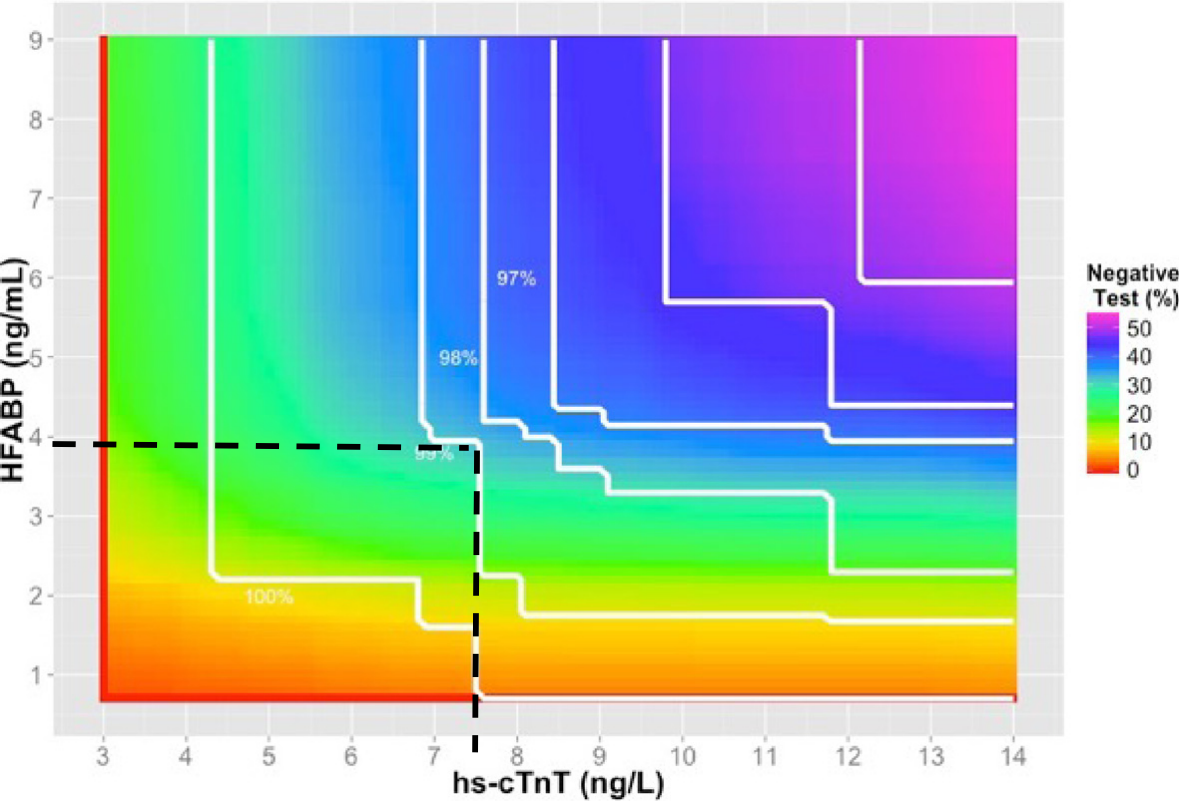
A Contour plot of the sensitivities (*white lines*) and heat map of the percentage of a negative test for AMI for any combination of H-FABP and hs-cTnT. Because multiple combinations of the two biomarkers can produce the same sensitivity we have plotted lines of constant sensitivity (eg 99 %). The colours represent the proportion of patients who have a negative test for each possible combination of biomarkers. *Red colours* represent few patients with negative tests, *purple colours* represent many patients with negative test. To focus on the high sensitivity areas we have limited the biomarker values displayed to ≤9 ng/mL for H-FABP and <14 ng/L for hs-cTnT. A negative test is defined when H-FABP and hs-cTnT concentrations are below the levels on the y and x axis respectively

The optimal thresholds that identified the maximum proportion of negative test patients whilst maintaining >99 % sensitivity for AMI was H-FABP 3.9 ng/mL with hs-cTnT threshold 7.6 ng/L, which (in combination with ECG findings) classified 32.1 % of patients with a negative test (Fig. 5; Table 4). There were 7 false negatives (97.2 % sensitivity) for the combination of H-FABP and hs-cTnT at their respective 99th percentiles with ECG and 34.2 % of patients were identified as negative for AMI (Table 4). The ROC derived optimal threshold for H-FABP combined with hs-cTnT ≥99th percentile was 3.0 ng/mL. Applying this threshold reduced the number of false negatives by 2 (98.0 % sensitivity) and the overall proportion of negative patients to 26.9 %.

**Table 4:** Performance of H-FABP with hs-cTnT and ECG

Test		AMI (*n* = 251)	No AMI (*n* = 834)	Total (*n* = 1085)	Proportion Negative Test (%)	Sensitivity (%)	NPV (%)
Optimal combination^a^ : ECG positive or hs-cTnT ≥7.6 ng/L or H-FABP ≥3.9 ng/mL	Positive	246	487	733		99.2 (97.1 to 99.8)	
	Negative	2	344	346	32.1		99.4 (97.9 to 99.8)
Hs-cTnT 99th percentile threshold: ECG positive or hs-cTnT ≥14 ng/L	Positive	235	253	488		94.8 (91.2 to 96.9)	
	Negative	13	578	591	54.8		97.8 (96.3 to 98.7)
Hs-cTnT or H-FABP 99th percentile threshold: ECG positive or hs-cTnT ≥14 ng/L or H-FABP ≥ 3.6 ng/mL	Positive	241	469	710		97.2 (94.3 to 98.6)	
	Negative	7	362	369	34.2		98.1 (96.1 to 99.1)
Hs-cTnT 99th percentile with H-FABP ROC derived^b^: ECG positive or hs-cTnT ≥ 14 ng/L or H-FABP ≥ 3.0 ng/mL	Positive	243	546	789		98.0 (95.4 to 99.1)	
	Negative	5	285	290	26.9		98.3 (96.0 to 99.3)
Hs-cTnT threshold for >99.0 % sensitivity: ECG or hs-cTnT ≥ 6.4 ng/L	Positive	247	431	678		99.6 (97.8 to 99.9)	
	Negative	1	400	401	37.2		99.8 (98.6 to 100.0)

^a^Strategy that yielded the maximum proportion of low-risk patients whilst maintaining a minimum sensitivity for AMI of 99 %

^b^H-FABP ROC derived threshold which maximized the combination of sensitivity and specificity in patients negative for hs-cTnT and ECG

### Subgroup analysis

From 1016 patients with a documented date and time of symptom onset, 215 (21 %) patients presented in <3 h, 273 (27 %) in 3–6 h, and 528 (52 %) after >6 h. Table 5 shows the sensitivity and proportion of negative test patients identified by the optimal combination of H-FABP and hs-cTn thresholds, according to duration from symptom onset to sample collection.

**Table 5:** Subgroup Analyses Performance of H-FABP with hs-cTn and ECG according to duration from symptom onset to sample collection

Hours from onset of symptoms to sample collection	Proportion of patients with documented date and time of symptom onset, n (%)	Optimal combination^a^ : ECG positive or hs-cTnI ≥10 ng/L or H-FABP ≥4.3 ng/mL		Optimal combination^a^ : ECG positive or hs-cTnT ≥7.6 ng/L or H-FABP ≥3.9 ng/mL	
		Sensitivity (%)	Proportion Negative Test Patients^b^	Sensitivity (%)	Proportion Negative Test Patients^c^
<3 h	215 (21)	100.0 (96.0 to 100.0)	43.3 %	100.0 (96.0 to 100.0)	34.4 %
3–6 h	273 (27)	100.0 (93.7 to 100.0)	43.2 %	98.2 (90.7 to 99.7)	34.4 %
>6 h	528 (52)	98.7 (95.3 to 99.6)	38.4 %	99.3 (96.3–99.9)	30.3 %

^a^Strategy that yielded the maximum proportion of low-risk patients whilst maintaining a minimum sensitivity for AMI of 99 %

^b^ *P*-value is 0.30 for comparison of proportion of negative test patients between subgroups (chi-square analysis)

^c^ *P*-value is 0.37 for comparison of proportion of negative test patients between subgroups (chi-square analysis)

### Additional analysis

Using the optimal combination of H-FABP and hs-cTnI or Hs-cTnT thresholds to rule-out index AMI, we estimated that the total number of bed days per patient could be reduced from 3.5 days to 2.7 days and 2.9 days respectively, providing potential hospital bed savings of up to 0.8 days (22 %) per presentation.

## Discussion

In high-risk patients requiring AMI rule-out, the addition of H-FABP to hs-cTnI at ED presentation, in the absence of new ischaemic changes on ECG may accelerate diagnostic decision making by identifying up to 40 % of such patients as low-risk for AMI on the basis of blood tests performed at presentation. When ECG, and H-FABP were combined with hs-cTnT, this multi-marker strategy ruled out AMI in fewer patients (32 %).

Rather than simply reporting the sensitivity with a 95 % confidence interval for a particular combination of H-FABP and hs-cTn, we deliberately, and conservatively, used a methodology to estimate the optimum combination of thresholds at high sensitivity (99 %) for AMI. This is important, because at such high sensitivities even large studies, such as this, will have large confidence intervals which limit general application of the results. Our multi-marker strategies utilizing the derivation of optimal thresholds for H-FABP, hs-cTn and ECG ie. thresholds that identified of the maximum proportion of negative patients whilst maintaining >99 % sensitivity, allowed the greatest proportion of patients to be classified as low risk (ie. negative test) with an acceptable sensitivity (ie. >99 %) for AMI detection at presentation. The use of strategies using pre-specified cut points, including the 99th percentile thresholds for either hs-cTn and 99th percentile or ROC-derived thresholds for H-FABP in combination with a positive ECG, improved the sensitivity for AMI in comparison to the 99th percentile thresholds for hsTn and ECG alone, but did not achieve >99 % sensitivity, considered an acceptable target by the majority of ED clinicians [25]. Utilizing the hs-cTnI LoD threshold in combination with a positive ECG resulted in no false negatives for AMI, but less than 10 % of patients could be classified as low-risk. In contrast, hs-cTnT at the LoD threshold in combination with a positive ECG, classified 30 % of patients as low-risk for AMI, which was only slightly less than utilizing the optimized rule-out strategy incorporating hs-cTnT, H-FABP and ECG (32.1 %). Subgroup analysis of our optimized biomarker strategies according to time from onset of symptoms showed that the classification of low-risk patients was not significantly greater in early presenters versus later presenters.

A number of studies have evaluated the utility of “at presentation” AMI rule-out strategies incorporating sensitive or hs-cTn assays in patients with suspected cardiac chest pain, but reported either unsatisfactory diagnostic accuracies [27], or insufficient identification of low-risk (negative test) patients suitable for early discharge [28, 29]. More recently, Shah and colleagues derived a threshold of 5 ng/L for hs-cTnI based on a target 99.5 % NPV in a cohort of patients without myocardial necrosis on presentation for the outcome of type 1 MI or cardiac death within 30 days [30]. We chose to use sensitivity for AMI rather than NPV because it is not affected by disease prevalence. Shah et al. observed a sensitivity of 98.9 % (95 % CI 97.9 to 99.4) for the primary outcome, and the proportion of patients with a negative test of 47.5 % in their derivation cohort [30]. Given the differences in AMI presentation rates in New Zealand the well-developed primary care system means many low risk patients do not present to EDs, it is difficult to compare directly the proportion of negative patients. Nevertheless, the proportion of negative test patients reported by Shah and colleagues is slightly greater than we observed with our optimized multi-marker strategy using hs-cTnI, but the sensitivity is slightly lower. A study of 14,612 presentations, found that up to 61 % of patients with initially undetectable hs-cTnT levels (5 ng/L) could be discharged directly from ED, with a NPV of 99.8 % (95%CI: 99.7 to 99.9) for AMI within 30 days, but with a sensitivity of only 98.1 % (95%CI: 96.9 to 98.8) [31].

Several established accelerated diagnostic pathways (ADPs) utilizing risk scores together with 0- and either 2- or 3 h cardiac troponin tests, have been shown to accurately identify up to 40 % of patients as at low-risk for MACE at 30 days in randomized controlled trials [5, 6]. We have previously reported that use of an ADP incorporating the Thrombolysis in Myocardial Infarction score, 0 h ECG, and 0- and 2-h troponin tests, enabled safe early discharge of almost double the proportion of patients compared to the standard care pathway (19.3 % versus 11.0 %, respectively), with a further 12.9 % of inpatients classified as low-risk although none ultimately were diagnosed with ACS [5]. Mahler and colleagues, showed that compared to usual care, implementation of the HEART Pathway, an ADP using the HEART [32] score with 0- and 3-h cardiac troponin tests), resulted in a significant reduction in objective cardiac testing and increase in early discharge without any MACE at 30 days (39.7 % versus 18.4 %, *P* < 0.001) in low-risk patients [6].

The strategy derived in this feasibility study, has the potential to improve the utility of AMI rule-out in the ED setting, as the test was negative in up to 40 % of patients on the basis of presentation blood sampling alone. The ability to safely identify a similar number of low-risk patients at presentation rather than after 2 h or more, currently required for established ADPs, will have likely benefits to both patients and the health care resources, since it is well established that prolonged assessment and ED overcrowding contribute to worse patient outcomes [7–9]. Furthermore, implementation of this strategy has a potential advantage over other ADPs in that it does not rely on expert clinical evaluation and comprehensive scoring systems that may be difficult to implement in routine clinical practice with less experienced clinicians or in overcrowded, busy ED departments. Nevertheless, the use of clinical scores in combination with our marker based strategy should be evaluated. Utilizing an “at presentation” AMI rule-out strategy may also have health cost benefits, as implied in the present study, with a reduction in bed days.

### Limitations

Our marker based strategies deliberately did not take into account clinical parameters, which should always be integrated into clinical decision-making. Incorporation of clinical assessment would potentially increase sensitivity, but may decrease the proportion of patients classified as low-risk (negative test). The underlying data are derived from a cohort with an AMI rate higher than most in the international literature and with relatively late presentation. This probably reflects effective pre-screening by the local primary health care system, reducing the proportion of very low-risk patients presenting to the ED. The current findings require validation in other ‘high risk’ and “lower cardiac risk” cohorts, and further analyses are underway. Compared to many ED populations, because of the well-funded primary health care system in New Zealand the prevalence of AMI was high. This means that translation of these optimal rule-out strategies to the general population (comprising more low-risk patients), would likely increase the NPV. Whether clinicians can effectively implement the optimized multi-marker strategies for on presentation rule-out of AMI in clinical practice and whether these strategies will improved discharge rates, is unknown. There may be reluctance by clinicians to adopt alternative rule-out strategies to those that utilize the hs-cTn 99th percentile threshold, despite this being a somewhat arbitrary and not sensitive cut-point to rule-out AMI [33]. Another issue is that below these thresholds the CV for these hs-cTn assays is much greater than 10 %. This would be even more of an issue for single hs-cTn strategies with ultra-low concentrations used to rule-out AMI. We note that when combining biomarkers to derive optimal thresholds the sensitivity contours were not perfectly smooth. This highlights the need for validation of the derived thresholds in other cohorts. It is important to note, that while the potential for cost savings may occur in patients classified as a negative test, with a reduction in hospital days, patients may still require cardiology investigations as part of their outpatient follow-up. We had limited power for the subgroup analyses, which found no significant effect of the optimized H-FABP multi-marker strategies, according to timing from symptom onset to ED admission blood sampling (>3 h, 3 to 6 h and >6 h). The proportion of patients classified with a negative test was, however, reduced when sample collection was greater than 6 h from onset of chest pain.

## Conclusions

In this study, H-FABP in combination with hs-cTn, for patients without ischemic ECG changes, improved the rule-out of AMI at ED presentation, compared to hs-cTn alone and ECG, while maintaining >99 % sensitivity for AMI. This is a potential strategy for implementation in clinical practice, and would allow up to 40 % of patients to be classified as low risk and suitable for early discharge and outpatient review. If implemented this strategy would likely have important consequences to patient flow within the ED and to hospital budgets, and patient morbidity and mortality.

## Abbreviations

ACS, acute coronary syndrome; ADP, accelerated diagnostic pathway; AMI, acute myocardial infarction; cTn, cardiac troponins; CV, coefficient of variation; ECG, electrocardiography; ED, Emergency Department; H-FABP, Heart Fatty Acid Binding Protein; hs-cTnI, high-sensitivity troponin I; hs-cTnT, high-sensitivity troponin T; LoD, limit of detection; MACE, major adverse cardiac events; ROC, Receiver Operator Characteristic curve; s-cTnI, sensitive cTnI; STEMI, ST segment elevation myocardial infarction
